# Comprehensive Triterpenoid Saponin Profiling of *Beta vulgaris* L. Root Extracts and Their Functional Application in Cleansing Gels

**DOI:** 10.3390/molecules31193480

**Published:** 2026-09-29

**Authors:** Bartosz Gierałt, Magdalena Anna Malinowska, Anna Tekieli-Galas, Mariusz Kowalczyk, Aneta Spórna-Kucab

**Affiliations:** 1 Department of Chemical Technology and Environmental Analysis, Faculty of Chemical Engineering and Technology, Cracow University of Technology, Warszawska 24, 31-155 Cracow, Poland; b.gieralt@op.pl (B.G.); anna.tekieli-galas@pk.edu.pl (A.T.-G.); 2 Department of Organic Chemistry and Technology, Faculty of Chemical Engineering and Technology, Cracow University of Technology, Warszawska 24, 31-155 Cracow, Poland; 3 Department of Biochemistry, Institute of Soil Science and Plant Cultivation, State Research Institute, Czartoryskich 8, 24-100 Puławy, Poland; mkowalczyk@iung.pulawy.pl

**Keywords:** triterpenoid saponins, *Beta vulgaris* L., biosurfactants, cleansing gels, UHPLC–CAD–QTOF–HRMS/MS, radical-scavenging activity

## Abstract

Plant-derived bioactive ingredients are increasingly valued in cosmetic formulations due to their multifunctionality and natural origin. This study evaluated beetroot (*Beta vulgaris* L.) extracts as plant-derived surfactant-supporting ingredients in cleansing gels, with DPPH radical-scavenging response assessed as a secondary formulation-level endpoint. Three cultivars—Dark Red Round (red), Boldor (yellow), and Snow Ball (white)—were characterized for triterpenoid saponin composition and functional performance. Unlike previous studies focused primarily on the phytochemical characterization of beetroot saponins, the present work integrates cultivar-dependent chemical profiling with comparative evaluation of the corresponding extracts in the same cleansing formulation. UHPLC–CAD–QTOF–HRMS/MS analysis tentatively annotated 30 triterpenoid saponins and revealed cultivar-dependent differences. Semi-quantitatively estimated total saponin concentrations were 17.5, 15.1, and 11.6 g/kg dry extract for Dark Red Round, Boldor, and Snow Ball, respectively, with oleanolic acid-based saponins predominating. Cleansing gels containing 1% (*w*/*w*) beetroot extract were evaluated for physicochemical, rheological, and functional properties. Extract-enriched gels showed increased foamability, cultivar-dependent foam stability, and pseudoplastic shear-thinning behavior. Surface tension remained substantially lower than that of water but increased moderately after incorporation of the extracts, indicating modification rather than further surface-tension lowering of the existing surfactant system. DPPH screening showed cultivar-dependent radical-scavenging responses, with the highest inhibition observed for the red beetroot formulation (8.94%). Overall, triterpenoid saponin-containing beetroot extracts modified the functional performance of the cleansing formulations, particularly their foaming and oil-emulsification properties. These findings support their potential as sustainable, plant-derived surfactant-supporting ingredients in multifunctional cleansing formulations.

## 1. Introduction

Plant-derived bioactive ingredients are gaining increasing attention in modern cosmetics due to their multifunctional biological activities, sustainability, and natural origin [1]. *Beta vulgaris* L. (beetroot) is a plant species valued not only for its culinary applications but also for its rich and diverse phytochemical composition. Beetroot contains numerous bioactive compounds, including pigments (betalains), polyphenols, flavonoids, vitamins, and triterpenoid saponins, which collectively contribute to its well-documented antioxidant, anti-inflammatory, and antimicrobial properties [2,3,4,5,6]. These characteristics make beetroot a promising candidate for cosmetic applications, particularly in functional cleansing formulations designed to maintain skin health [7,8].

Among the various bioactive constituents of beetroot, saponins play a particularly important role in cosmetic applications. Due to their amphiphilic molecular structure, comprising a hydrophobic aglycone and hydrophilic sugar moieties, saponins exhibit surfactant-like properties, including foaming, emulsifying, and cleansing activities, while some plant-derived saponins have been reported to show lower irritation potential than selected conventional surfactants, depending on their structure, source, concentration, and formulation context [9,10,11]. As a result, saponin-containing plant extracts are increasingly explored as naturally derived surface-active ingredients in cleansing formulations [12,13]. In addition to their surface-active properties, saponins have been reported to exhibit antioxidant and anti-inflammatory effects, further supporting their potential multifunctional role in cosmetic products [14,15].

It is also important to note that beetroot cultivars differ significantly in their pigmentation and bioactive compound profiles. Red beetroot varieties are particularly rich in betacyanins, yellow cultivars predominantly contain betaxanthins, while white beetroot varieties exhibit lower pigment content but may still retain notable antioxidant and surface-active potential. Cultivar-dependent differences may also involve the qualitative and quantitative composition of triterpenoid saponins, thereby influencing the functional properties of the resulting extracts. The intense natural coloration of red and yellow beetroots also offers potential for the development of naturally colored cosmetic formulations, although the stability of betalain-derived color depends strongly on formulation and storage conditions. This property aligns well with the clean beauty movement and growing consumer demand for sustainable, naturally derived ingredients in personal care products [16,17].

Cleansing cosmetics, such as facial and body wash gels, are designed to remove dirt, excess sebum, sweat, and environmental pollutants from the skin surface. Their effectiveness largely depends on the presence of surfactants, which facilitate impurity removal through emulsification and foam formation [18,19,20]. However, widely used synthetic surfactants, including sodium lauryl sulfate (SLS) and sodium laureth sulfate (SLES), have been associated with adverse skin effects, including dryness, irritation, and disruption of the epidermal barrier [21,22]. These concerns have intensified the search for milder and biocompatible surfactant systems. In this context, beetroot-derived saponins represent a promising source of naturally derived surface-active compounds that may support the performance of cleansing surfactant systems.

Previous studies have established that the qualitative and quantitative composition of triterpenoid saponins varies among *Beta vulgaris* cultivars and plant tissues [23,24,25,26,27]. Our previous work characterized triterpenoid saponins in the peel and flesh of differently colored beetroot cultivars using LC–ESI–MS/MS [23], whereas Mroczek et al. [24] applied UHPLC–CAD–QTOF–MS/MS to saponins occurring in Swiss chard leaves. However, these investigations were primarily focused on phytochemical characterization, and substantially less is known about how the composition of beetroot root extracts translates into their functional behavior after incorporation into cosmetic cleansing formulations. In particular, it remains unclear whether cultivar-dependent differences in saponin abundance and composition are directly reflected in formulation performance or whether the observed effects result from more complex interactions between individual saponins, co-extracted constituents, and the existing surfactant system [28,29,30].

Accordingly, the present study extends previous phytochemical investigations [23,24,25,26,27] by integrating accurate-mass UHPLC–CAD–QTOF–HRMS/MS profiling of triterpenoid saponins from three differently colored beetroot root extracts with comparative evaluation of their effects in the same model cleansing formulation. The extracts were incorporated at the same concentration and assessed with respect to foamability and foam stability, oil emulsification performance, surface tension, rheological behavior, water dispersibility, and radical-scavenging response. Thus, rather than focusing solely on the occurrence and structural diversity of beetroot saponins, the present work investigates whether and to what extent chemically distinct beetroot extracts influence selected physicochemical and functional properties of an existing cleansing system. This design permits evaluation of whether formulation-level responses reflect differences in extract composition without assuming a direct correspondence between saponin abundance and macroscopic formulation performance. The overarching aim was to determine whether triterpenoid saponin-containing beetroot extracts could modify selected functional properties of an existing cleansing surfactant system and thereby show potential as multifunctional plant-derived surfactant-supporting ingredients, with DPPH radical-scavenging response considered as a secondary formulation-level endpoint.

## 2. Results and Discussion

### 2.1. Tentative Annotation and Semi-Quantitative Analysis of Triterpenoid Saponins

**Figure 1:**
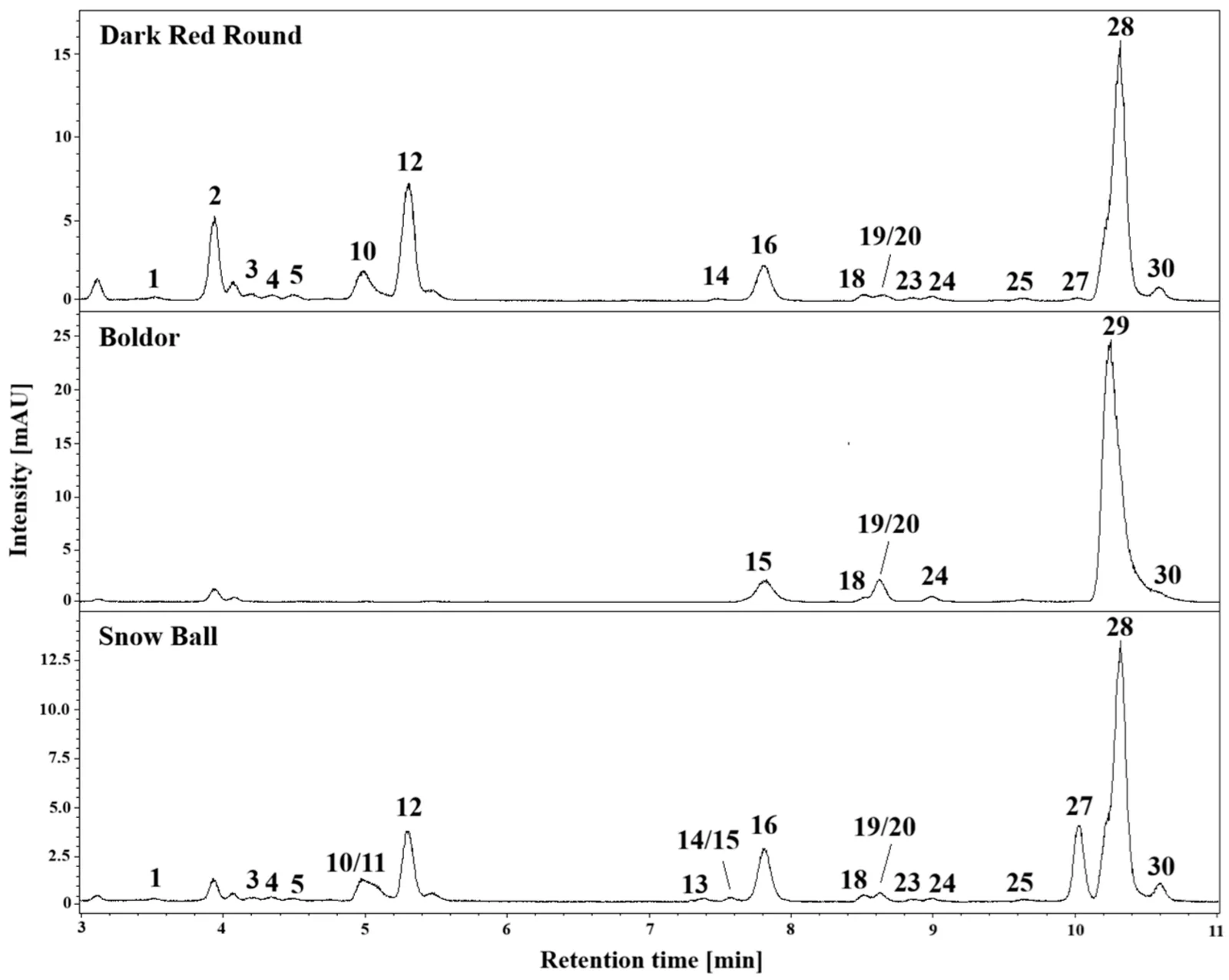
UHPLC–CAD chromatographic profiles of saponin-rich extracts obtained from *Beta vulgaris* L. root cultivars: Dark Red Round, Boldor, and Snow Ball. Peaks are numbered according to the tentatively annotated saponins listed in Table 1.

In the present study, a total of 30 triterpenoid saponins were tentatively annotated in beetroot extracts obtained from three *Beta vulgaris* L. cultivars: Dark Red Round (red beetroot), Boldor (yellow beetroot), and Snow Ball (white beetroot), using UHPLC–CAD–QTOF–HRMS/MS. Compound annotation was based on accurate mass measurements (mass error <5 ppm), characteristic MS/MS fragmentation patterns, and comparison with previously reported spectral data. Several of the detected compounds were annotated as betavulgarosides (I–IX) (Figure 1 and Table 1).

The structural diversity of the identified saponins resulted from variations in both the aglycone core and glycosylation patterns. The majority of saponins were derived from oleanolic acid, a pentacyclic triterpenoid aglycone known for its anti-inflammatory and skin-protective properties [31,32]. Additional aglycones, including hederagenin and akebonoic acid, were also identified, further demonstrating the structural diversity of triterpenoid saponins present in *B. vulgaris* extracts [23,24,25].

As previously reported [26,27], the complexity of *B. vulgaris* saponins is further enhanced by the number and nature of substituents attached to the aglycone core. In addition to common sugar moieties such as hexose (Hex), pentose (Pen), and uronic acid (UrA), less typical functional groups—including dioxolane (Diox) and acetal (Act)—were observed. Notably, up to four substituents per molecule were identified, resulting in a broad range of deprotonated molecular ions (*m*/*z* 615.3538–1117.5055). These results demonstrate the considerable structural diversity of triterpenoid saponins present in the analyzed beetroot extracts.

Representative HRMS/MS fragmentation pathways were examined to further support the tentative annotation of acetal- and dioxolane-containing saponins. For Diox–Hex–UrA–oleanolic acid (compound 10, [M–H]^−^ at *m*/*z* 953.4382), fragmentation produced an ion at *m*/*z* 793.4378, corresponding to a neutral loss of 160.0004 Da, consistent with a dioxolane-type substituent. Subsequent product ions at *m*/*z* 631.3846 and 455.3529 were consistent with the consecutive losses of a hexose residue (162.0532 Da) and uronic acid (176.0317 Da), respectively, yielding the characteristic oleanolic acid aglycone ion. In comparison, Act–Hex–UrA–oleanolic acid (compound 12, [M–H]^−^ at *m*/*z* 955.4543) generated the product ion at *m*/*z* 793.4380 through a neutral loss of 162.0163 Da, consistent with an acetal-type substituent, followed by ions at *m*/*z* 631.3851 and 455.3532. Comparable diagnostic fragmentation was observed for Act–UrA–oleanolic acid (compound 28; *m*/*z* 793.4005 → 631.3842 → 455.3526) and Diox–UrA–oleanolic acid (compound 29; *m*/*z* 791.3851 → 631.3839 → 455.3519). These fragmentation patterns are consistent with the detailed HRMS/MS characterization of *B. vulgaris* saponins reported previously [33] and provide additional support for differentiation of acetal- and dioxolane-substituted derivatives.

Semi-quantification of saponins in the dried beetroot extracts was performed using a UHPLC–CAD method adapted from Mroczek et al. [24,33]. Due to variability in detector response and sensitivity limitations, only the major saponins were quantified using external reference standards. Accordingly, the reported concentrations are primarily intended for comparative evaluation of saponin abundance among cultivars and should not be interpreted as definitive absolute concentrations of individual betavulgarosides. The calibration curves showed R^2^ values > 0.996, with LOQ values of 6.7–7.5 mg/kg dry extract (Table 2).

Semi-quantitative analysis revealed pronounced cultivar-dependent differences in total saponin content. The Dark Red Round cultivar exhibited the highest total saponin concentration, reaching 17,455 ± 742.0 mg/kg dry extract (DE), followed by Boldor (15,119 ± 1039.5 mg/kg DE) and Snow Ball (11,623 ± 578.9 mg/kg DE). In Dark Red Round, saponin 28 (*m*/*z* 793.4005) was the predominant compound, with a concentration of 8447.1 ± 675.8 mg/kg DE, indicating marked cultivar-dependent accumulation of this saponin.

A previous report [23] identified Boldor as one of the richest beetroot cultivars in terms of saponin content, with saponin 28 (Act–UrA–oleanolic acid) reported as the dominant compound (7361 ± 117 mg/kg fresh weight). In contrast, in the present study, saponin 28 was not detected in Boldor above the limit of quantification, while saponin 29 (Diox–UrA–oleanolic acid) was identified as the predominant compound, reaching 12,989 ± 1039.1 mg/kg DE. Although these values cannot be directly compared because they are expressed on a fresh-weight and dry-extract basis, respectively, the differences in the predominant saponin profile may reflect variation in plant material, extraction conditions, or sample processing.

Although 50% aqueous acetone provided comparable extraction yields among the three cultivars, its extraction efficiency and selectivity were not directly compared with alternative extraction approaches. Greener techniques, such as ultrasound-assisted or pressurized liquid extraction, may reduce solvent consumption and extraction time and should be considered in future optimization studies.

Snow Ball (white beetroot) cultivar exhibited the lowest total saponin content among the analyzed samples. Nevertheless, measurable levels of saponins 12 (1351.6 ± 81.1 mg/kg DE), 16 (1181.3 ± 70.9 mg/kg DE), and 28 (6974.0 ± 557.9 mg/kg DE) were detected, demonstrating that the white cultivar also contained substantial amounts of selected triterpenoid saponins (Figure 1, Table 1 and Table 2).

Thus, the present study extends our previous phytochemical investigation [23] beyond the description of cultivar-dependent saponin composition. The chemically differentiated extracts characterized here were incorporated at the same concentration into the same cleansing formulation, allowing direct comparison of their formulation-level effects under identical experimental conditions. As discussed below, the resulting functional responses did not uniformly parallel total saponin abundance, indicating that formulation performance did not show a simple correspondence with total saponin content and may also depend on qualitative differences in saponin profiles, co-extracted constituents, and interactions with the existing surfactant system.

### 2.2. Physicochemical and Functional Properties of Beetroot-Enriched Cleansing Gel Formulations

#### 2.2.1. pH of the Cleansing Formulations

The surface of human skin is naturally protected by an acidic mantle, with a typical pH ranging from 4.5 to 5.5. This mildly acidic environment is essential for maintaining epidermal barrier integrity, regulating the activity of enzymes involved in lipid synthesis, and supporting a balanced skin microbiota [34,35,36]. Disruption of the acid mantle, particularly by cleansing products with an alkaline pH, has been associated with increased transepidermal water loss (TEWL), impaired barrier function, skin dryness, irritation, and increased susceptibility to microbial imbalance and inflammation [37]. Consequently, maintaining an appropriate pH is a critical requirement in the formulation of skin-cleansing products, especially those intended for frequent use or application on sensitive skin.

In this context, the pH values of the base and beetroot-enriched cleansing gel formulations were evaluated (Table 3). The base gel exhibited a pH of 6.23 ± 0.02. Incorporation of 1% (*w*/*w*) beetroot extracts resulted in a moderate decrease in pH, yielding values of 5.39 ± 0.01 for the gel containing yellow beetroot extract, 5.30 ± 0.02 for the formulation with white beetroot extract, and 5.42 ± 0.01 for the gel enriched with red beetroot extract.

The decrease in pH observed after incorporation of the beetroot extracts reflects the overall contribution of extract-derived constituents; however, the individual compounds responsible for this effect were not identified. The final pH values of the beetroot-enriched formulations remained within the mildly acidic range relevant to skin-cleansing products. Long-term chemical and physical stability of the complete formulations was not evaluated in the present study; therefore, formulation stability cannot be inferred from pH values alone.

Color stability was not evaluated during storage. Therefore, the observed beetroot-derived coloration should be regarded as an initial formulation attribute rather than evidence of long-term color stability. Dedicated storage studies would be required to assess the stability of the formulations over time.

#### 2.2.2. Oil Emulsification Performance

The primary function of cleansing formulations is to effectively remove oily impurities, sebum, and particulate contaminants from the skin surface. This process depends on surfactants’ ability to emulsify hydrophobic substances and stabilize oil–water interfaces, thereby facilitating their removal during rinsing [38,39]. Although consumers often perceive foaming as an indicator of cleansing efficacy, detergency is more closely related to the surfactant system’s emulsification capacity and interfacial activity [40,41].

In the present study, oil emulsification and removal performance was evaluated using a visual, semi-quantitative assay based on changes in emulsion appearance and scored on a seven-level ordinal scale ranging from 0 to 6 (Table 3). The base gel without plant extract received a score of 0, with visible oil separation and poor emulsification. In contrast, all formulations containing beetroot extracts achieved the maximum score (6), indicating effective emulsification of the oily phase and formation of homogeneous oil–water systems without a visible oil ring under the applied test conditions.

The enhanced emulsification performance of beetroot-enriched gels is consistent with the known surface-active properties of triterpenoid saponins. However, because sodium lauroyl sarcosinate was present at the same concentration in all formulations, the individual contribution of beetroot-derived saponins cannot be separated from that of the primary surfactant under the present experimental conditions. Because all formulations contained the same concentration of sodium lauroyl sarcosinate, the present study was designed to evaluate whether beetroot extracts could modify the functional behavior of an existing cleansing surfactant system rather than to assess their performance as standalone surfactants. The observed increases in foamability and oil-emulsification performance therefore support a surfactant-supporting role of the extracts under the applied formulation conditions.

The extract-free base formulation containing 7.0% (*w*/*w*) sodium lauroyl sarcosinate served as the internal surfactant control in the present study, allowing the effect of beetroot extract incorporation to be evaluated within an otherwise identical formulation matrix. A separate benchmark formulation based on SLS, SLES, or alkyl glucosides was not prepared. Therefore, direct experimental ranking against these commonly used surfactants cannot be made from the present data. Nevertheless, published studies provide useful external context. Sodium lauroyl sarcosinate has been shown to exhibit substantial surface activity and foaming performance in personal-care surfactant systems, while SLES and alkyl glucosides such as coco- and decyl glucoside show distinct foaming, surface-tension, and cleansing behaviors depending on formulation composition and test conditions [42,43,44]. Consequently, the literature comparisons should be regarded as qualitative benchmarks rather than direct numerical comparisons with the present formulations.

Saponins possess amphiphilic molecular structures composed of hydrophobic aglycones and hydrophilic sugar moieties, enabling efficient adsorption at oil–water interfaces and promoting emulsification [28,45,46]. Numerous studies have demonstrated that saponin-rich plant extracts can improve emulsification and cleansing-related properties by lowering interfacial tension and stabilizing oil-in-water emulsions [28,29]. Comparable effects have been reported for saponin extracts from *Sapindus mukorossi*, *Camellia oleifera*, and *Acacia concinna* [47,48,49]. Importantly, plant-derived saponins have been reported to exhibit lower irritation potential than some conventional synthetic surfactants, depending on their source, composition, and concentration [50]. However, skin irritation, cytotoxicity, and dermatological compatibility of the final beetroot-enriched formulations were not directly evaluated in the present study. Therefore, the mildness of the tested formulations cannot be concluded from the physicochemical and functional results alone. Sodium lauroyl sarcosinate is widely used as a cleansing surfactant and has been reported to show low irritation potential in cosmetic applications [51]. Nevertheless, the mildness of a complete cleansing formulation depends on the combined effects of surfactant composition, concentration, pH, and interactions with the stratum corneum, and cannot be inferred from pH alone [52]. Dedicated skin-compatibility studies, such as validated reconstructed human epidermis irritation testing or controlled clinical application/patch testing, would therefore be required to evaluate the irritation potential and dermatological compatibility of the final formulations [52,53].

#### 2.2.3. Rheological Properties

In addition to cleansing efficacy, rheological behavior plays a crucial role in the performance, stability, and consumer acceptance of topical gel formulations, as it directly affects ease of dispensing and application, as well as sensory perception during use [54,55,56]. Rheological measurements were therefore conducted to evaluate the influence of saponin-rich beetroot extracts on the flow behavior of the cleansing gels.

**Figure 2:**
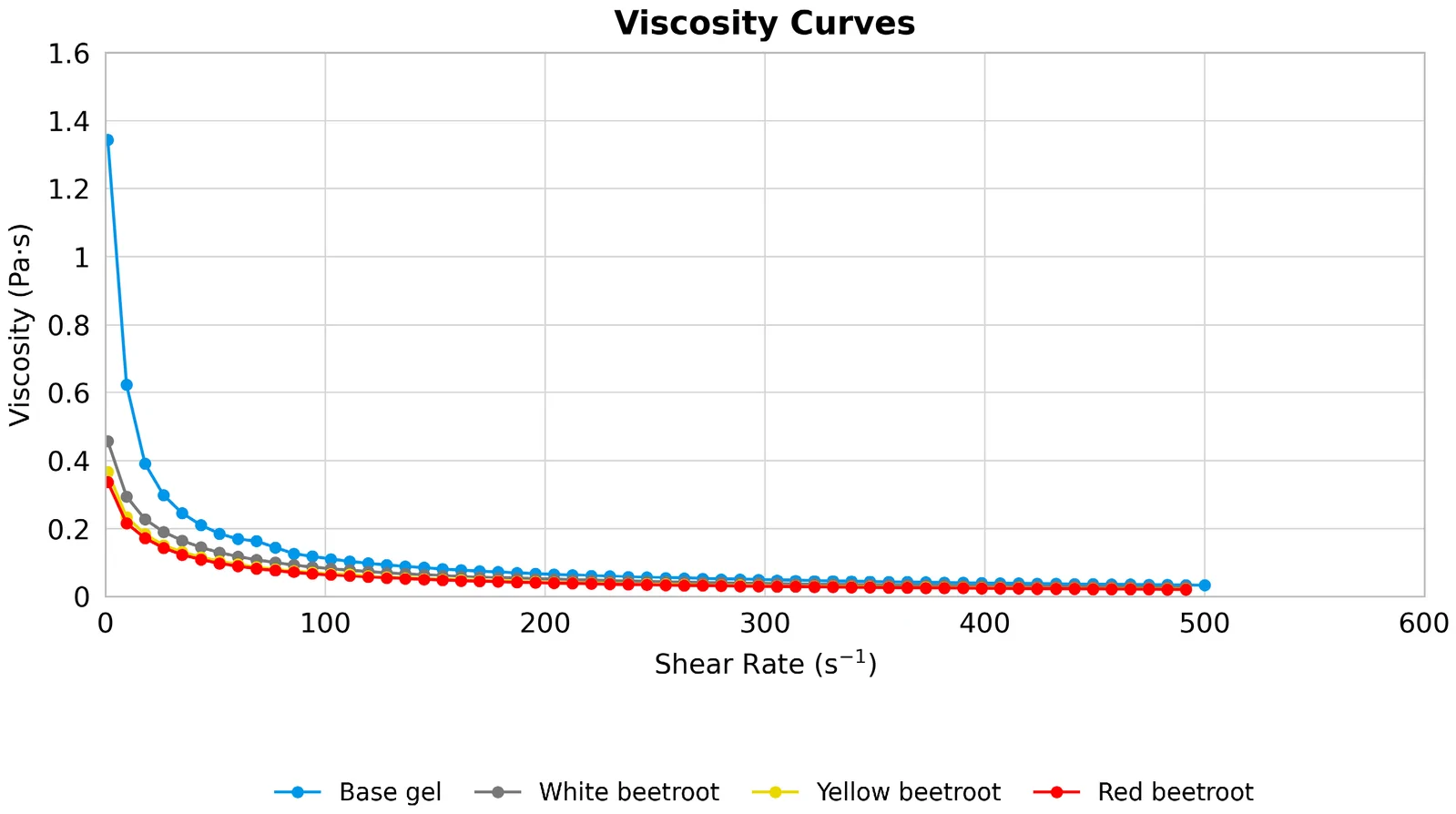
Apparent viscosity as a function of shear rate (1–500 s^−1^) for the base cleansing gel and gels containing 1% (*w*/*w*) beetroot extracts (white, yellow, and red cultivars). All formulations exhibited pronounced shear-thinning behavior characteristic of pseudoplastic, non-Newtonian fluids.

Figure 2 presents viscosity curves showing apparent viscosity as a function of shear rate (1–500 s^−1^). All investigated formulations exhibited pronounced shear-thinning behavior, typical of pseudoplastic, non-Newtonian fluids used in cosmetic gels [54,57]. This behavior reflects progressive disruption and alignment of the internal gel structure under applied shear stress [58].

The base gel displayed the highest apparent viscosity, whereas beetroot-enriched gels exhibited slightly lower viscosity values. Similar viscosity reductions after incorporation of plant extracts have been reported previously and may result from interactions between low-molecular-weight phytochemicals and the polymeric gel network [59]. Minor differences among beetroot cultivars may be related to differences in saponin composition and the presence of co-extracted compounds [28].

**Figure 3:**
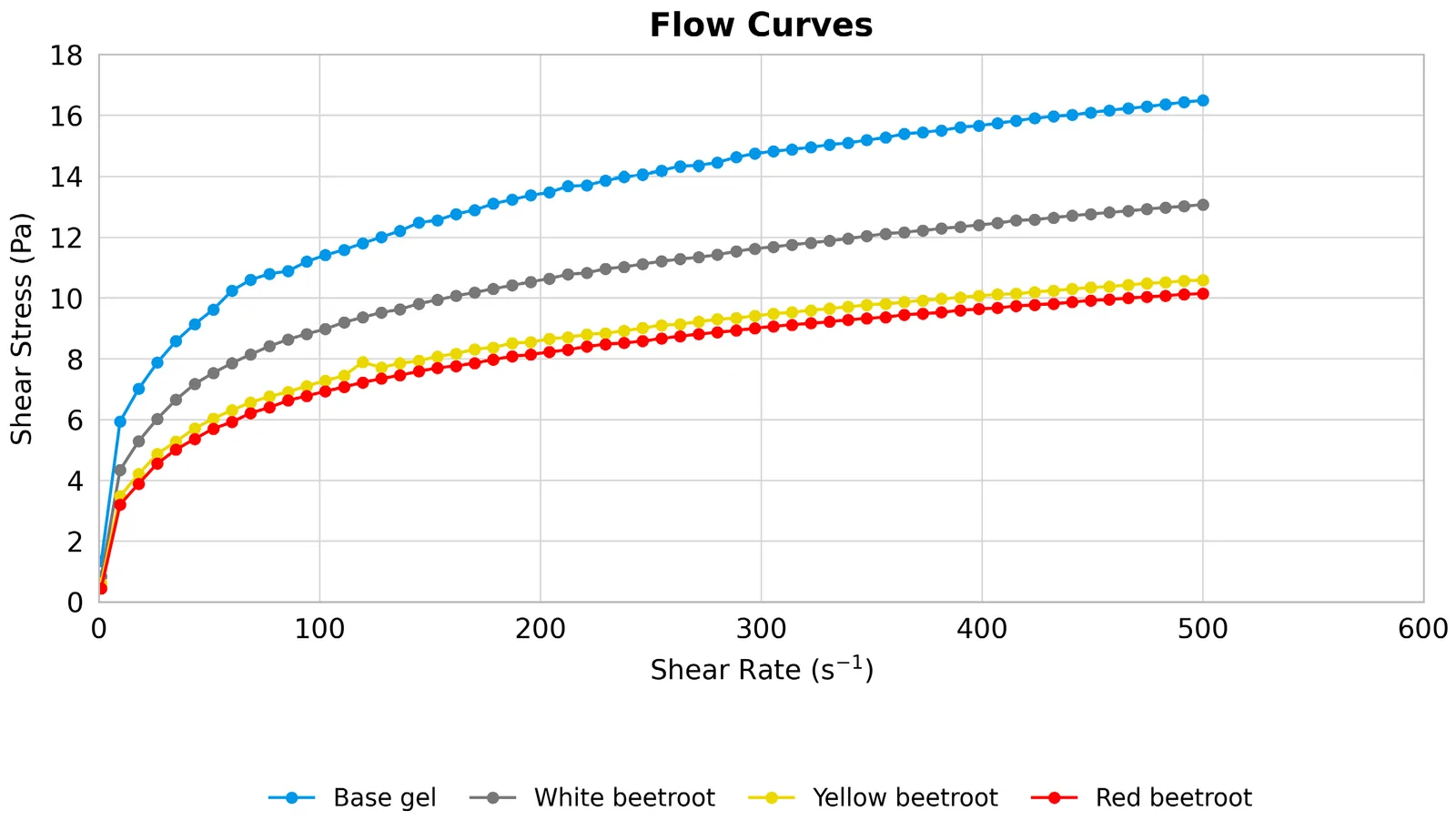
Flow curves (shear stress versus shear rate) of the base gel and beetroot-enriched cleansing gels, confirming non-Newtonian behavior and showing that incorporation of saponin-rich beetroot extracts did not markedly alter the overall flow characteristics of the formulations.

Flow curves (shear stress versus shear rate) shown in Figure 3 further confirmed non-Newtonian behavior. The overall similarity of the flow curves indicates that incorporation of beetroot extracts did not markedly alter the characteristic flow behavior of the gel formulations under the applied measurement conditions.

#### 2.2.4. Water Dispersibility Test

Water dispersibility was assessed by measuring the time required for the cleansing gel to form a visually uniform dispersion in water under standardized mixing conditions (Table 3) [54]. The base gel without plant extract exhibited the shortest dispersion time (3.40 ± 0.11 min), whereas incorporation of beetroot extracts increased the time required to achieve uniform dispersion. The white and yellow beetroot formulations showed dispersion times of 5.52 ± 0.08 and 5.45 ± 0.07 min, respectively, while the red beetroot formulation showed an intermediate value of 4.65 ± 0.64 min.

Although the beetroot-enriched gels exhibited lower apparent viscosity than the base formulation, dispersion time did not follow the same trend. This indicates that water dispersibility under the applied conditions was not governed by bulk viscosity alone and may also depend on formulation structure and interactions among individual components. Apparent viscosity measured under steady shear characterizes the flow response of the intact formulation, whereas dispersion in water additionally requires water penetration into the gel, local dilution, disruption and rearrangement of the formulation structure, and breakup of the gel into smaller domains. Consequently, a lower steady-shear viscosity does not necessarily imply faster dispersion in water. The beetroot extracts may have altered local interactions within the formulation matrix without increasing the steady-shear apparent viscosity.

Therefore, the present test should be regarded as a comparative assessment of water dispersibility rather than as a direct measure of rheological behavior. Yield stress and thixotropy were not determined in the present study; therefore, their potential relationship with dispersion behavior could not be assessed.

#### 2.2.5. Surface Tension

Surface tension is a key parameter reflecting the interfacial activity of surfactants and directly influences both detergency and foam formation in cleansing formulations [39,60]. Surface tension measurements performed using the du Noüy ring method were used to evaluate changes in the interfacial properties of the formulations following incorporation of beetroot extracts (Table 3).

The base gel exhibited a surface tension value of 35.34 ± 0.25 mN/m, while incorporation of beetroot extracts resulted in a moderate increase in surface tension. Gels containing yellow, white, and red beetroot extracts showed surface tension values of 39.66 ± 0.03 mN/m, 41.84 ± 0.05 mN/m, and 41.29 ± 0.07 mN/m, respectively. For comparison, distilled water had a surface tension of 70.17 ± 0.06 mN/m, which was considerably higher than that of the other samples.

Although the beetroot-enriched formulations exhibited higher surface tension values than the base gel, all values remained substantially lower than that of distilled water, confirming the overall surface-active character of the cleansing formulations. The observed differences indicate that incorporation of beetroot extracts modified the interfacial properties of the existing surfactant system rather than producing an additional reduction in surface tension.

Because sodium lauroyl sarcosinate was maintained at the same concentration (7.0% *w*/*w*) in all formulations, the observed increase in surface tension cannot be attributed to dilution of the primary surfactant. Rather, it may reflect interactions between the surfactant and extract-derived constituents, as well as changes in the physicochemical environment of the formulation after extract incorporation. Therefore, the present results do not demonstrate a synergistic surface-tension-lowering effect between beetroot-derived constituents and sodium lauroyl sarcosinate.

Importantly, the surface tension results should be considered together with the enhanced foamability and emulsification performance of the beetroot-enriched gels. Thus, although incorporation of the extracts did not further reduce surface tension, it modified the overall functional behavior of the surfactant system. Because the formulations contained sodium lauroyl sarcosinate, the individual contribution of beetroot-derived saponins cannot be separated from that of the synthetic surfactant under the present experimental conditions.

#### 2.2.6. Foaming Properties

Foaming properties were evaluated as an important functional parameter of cleansing formulations. Foam generated by mechanical agitation is a thermodynamically metastable system and, although foam formation is not a direct measure of detergency, it is widely used as an indicator of cleansing performance and consumer perception [40]. Surfactants facilitate foam formation by reducing the air–liquid interfacial tension and promoting the incorporation of air into the liquid phase [61].

Incorporation of saponin-rich beetroot extracts markedly increased foamability compared with the base gel, with all beetroot-enriched formulations exhibiting identical foamability values of 60%, compared with 20% for the base gel. This pronounced increase indicates that the beetroot extracts contributed to the foaming performance of the existing surfactant system and is consistent with the known surface-active properties of triterpenoid saponins [30,46]. The lack of differences in foamability among the beetroot cultivars indicates that the overall foam-forming capacity was similar for all three extracts under the applied test conditions.

The absence of cultivar-dependent differences in foamability and visual oil-emulsification scores does not necessarily indicate identical surface-active behavior of the three extracts. Under the applied formulation conditions, these endpoints may have had limited discriminatory capacity, particularly because all extract-containing formulations reached the maximum score in the visual oil-emulsification assay. In addition, interactions between beetroot-derived constituents and the existing surfactant system may have reduced the expression of more subtle cultivar-dependent differences at the macroscopic level [62]. Therefore, identical foamability and oil-emulsification scores should be interpreted cautiously and do not exclude differences in the intrinsic interfacial behavior of the extracts.

In contrast, foam stability varied among the beetroot-enriched gels, with the order: red beetroot extract (90%) > yellow beetroot extract (83%) > white beetroot extract (67%). Notably, these values were lower than the 100% foam stability observed for the base formulation. The differences among beetroot-enriched formulations suggest that foam stability may depend on the qualitative composition of the extracts, including differences in saponin profiles and co-extracted constituents, which may influence interfacial film properties and foam drainage [29,30].

The simultaneous increase in foamability and decrease in foam stability is not contradictory, because foam formation and foam persistence are governed by different physicochemical factors [61,63]. The surface-active constituents of the beetroot extracts may facilitate initial foam formation, whereas foam stability additionally depends on liquid drainage, bulk viscosity, and the properties of the interfacial film surrounding the air bubbles [61,63]. The lower apparent viscosity of the extract-containing formulations may promote faster drainage from the foam lamellae and thus contribute to their lower stability relative to the base gel [63]. However, viscosity alone cannot explain the cultivar-dependent differences, because the red beetroot formulation exhibited the lowest apparent viscosity but the highest foam stability among the extract-containing formulations. Moreover, the moderate increase in equilibrium surface tension after extract incorporation does not contradict the increased foamability, since foam formation under dynamic agitation depends not only on equilibrium surface tension but also on adsorption kinetics and interfacial film formation [63,64]. The higher equilibrium surface tension of the extract-containing formulations may also reflect altered interfacial adsorption or packing and could contribute to reduced foam persistence; however, equilibrium surface tension alone is insufficient to predict foam stability, which also depends on drainage and the mechanical properties of the interfacial film [63,64,65].

Because the beetroot extracts are chemically complex, co-extracted constituents, including phenolic compounds and pigments, may also modify the bulk and interfacial environment of the formulations, although their individual contributions to foam behavior were not quantified in the present study. Moreover, mixed systems containing triterpenoid saponins and conventional surfactants can exhibit non-ideal and competitive adsorption at the air–water interface, indicating that the behavior of saponins in a multicomponent cleansing system may differ from that observed for isolated saponins [62].

The observed differences in foam stability cannot be attributed to aglycone type alone, as oleanolic acid derivatives predominated across the extracts. Interestingly, red and white beetroot extracts both contained high levels of Act–UrA–oleanolic acid derivatives but differed markedly in foam stability. This suggests that differences in glycosylation and substitution patterns may influence molecular amphiphilicity, interfacial adsorption, and the organization of the interfacial film [30,65]. However, because the extracts contain complex mixtures of saponins and other co-extracted constituents, these structure–function relationships should be regarded as exploratory rather than causal.

Recent studies with structurally defined triterpenoid saponins support this interpretation. Bajcura et al. [66] showed that hederacoside C and hederasaponin B, which differ only by a hydroxyl group at C-23 of the aglycone, exhibit markedly different critical micelle concentrations, demonstrating that relatively subtle changes in aglycone structure can modify surface-active behavior [66]. More recently, Wang et al. [67] demonstrated that glycosylation topology strongly affects solubility, micellization, and aggregate morphology: monodesmosidic α-hederin and hederacolchiside A1 exhibited substantially lower critical micelle concentrations than bidesmosidic hederacoside C [67]. These findings indicate that both aglycone structure and the number and position of sugar chains can substantially influence the self-assembly and interfacial behavior of triterpenoid saponins, supporting the interpretation that qualitative differences in saponin profiles may contribute to the cultivar-dependent formulation responses observed here.

Triterpenoid saponins are known to adsorb at air–liquid interfaces and form viscoelastic interfacial films; however, their foaming and stabilizing properties strongly depend on molecular structure and botanical origin [29,30,65]. Comparable behavior has been reported for saponins from *Camellia oleifera* [68].

### 2.3. DPPH Radical-Scavenging Activity of Beetroot-Enriched Cleansing Gel Formulations and Extracts

The DPPH radical-scavenging activity of the cleansing gel formulations and the corresponding beetroot extracts was evaluated using sample-specific blanks to correct for baseline intrinsic absorbance at 517 nm. The extract-free base gel, analyzed as a formulation-matrix control, exhibited negligible DPPH radical-scavenging activity.

Incorporation of beetroot extracts resulted in cultivar-dependent DPPH radical-scavenging responses compared with the base gel. The gel containing yellow beetroot extract exhibited only a negligible effect (0.05% inhibition), whereas formulations enriched with white and red beetroot extracts showed inhibition values of 3.78% and 8.94%, respectively. The highest DPPH radical-scavenging response was observed for the gel containing red beetroot extract (Table 4).

The observed differences among the formulations may be related to cultivar-dependent variations in phytochemical composition. Red beetroot is particularly rich in betalains, including betacyanins and betaxanthins, which are known to exhibit radical-scavenging activity [15,69]. White and yellow beetroot cultivars differ in pigment content and composition, which may partly explain the lower DPPH inhibition observed for these formulations [70,71].

To further investigate whether the gel matrix affected the measured antioxidant activity, the DPPH radical-scavenging activity of the corresponding beetroot extracts was determined at the same concentrations as those used in the gel formulations. The DPPH inhibition values obtained for the extracts were 2.01 ± 0.21%, 0.09 ± 0.02%, and 6.81 ± 0.47% for white, yellow, and red beetroot, respectively, compared with 3.78 ± 0.39%, 0.05 ± 0.04%, and 8.94 ± 0.87% for the corresponding gel formulations. Significant cultivar-dependent differences were observed within both the gel formulations and the corresponding extracts (one-way ANOVA, *p* < 0.001). Tukey’s HSD post hoc test showed that all three cultivars differed significantly from one another within each sample type (*p* < 0.05). In both the gel formulations and the extracts, red beetroot exhibited the highest DPPH radical-scavenging activity, followed by white beetroot, whereas yellow beetroot showed the lowest activity. The consistent ranking of the cultivars in both sample types indicates that the observed DPPH response was strongly associated with beetroot cultivar. Comparison with the corresponding extracts at equivalent concentrations therefore provides a direct reference for interpreting the formulation-level DPPH response.

Additionally, beetroot extracts contain phenolic compounds and saponins, which may also contribute to the observed antioxidant response [23]. However, because total phenolic content and betalain concentrations were not quantified in either the extracts or the final formulations, the relative contributions of saponins, betalains, phenolic compounds, and other phytochemicals to the observed DPPH response cannot be resolved. Accordingly, the DPPH radical-scavenging response observed in the present study should be attributed to the overall phytochemical composition of the beetroot extracts rather than specifically to triterpenoid saponins. The relatively low inhibition values observed in the present study may reflect both the low extract concentration (1% *w*/*w*) and interactions within the complex gel matrix, which can affect the accessibility of antioxidant constituents to the DPPH radical. Similar matrix-related effects have been reported for cosmetic formulations containing plant extracts [72].

The DPPH responses observed in the present formulations were modest, with the highest inhibition (8.94%) observed for the red beetroot formulation. These results demonstrate a measurable but modest DPPH radical-scavenging response in the final formulations. Plant-based cleansing formulations reported in the literature have also shown measurable DPPH activity; however, direct benchmarking is difficult because antioxidant performance is frequently reported using different assay conditions, sample concentrations, and endpoints. For example, published plant-based cleansing formulations have reported DPPH activity as IC_50_ values, whereas the present study determined percentage inhibition under a single assay condition [73,74]. Consequently, these literature data provide qualitative context but are not directly comparable with the values obtained in the present study. The antioxidant activity of the investigated formulations was evaluated using the DPPH radical-scavenging assay. Although complementary antioxidant assays, such as FRAP, ABTS, ORAC or photochemiluminescence, could provide additional information on the antioxidant potential of the tested samples, the present study focused on the DPPH assay. Therefore, the obtained results should be interpreted specifically in terms of DPPH radical-scavenging activity and should not be considered as a comprehensive characterization of the overall antioxidant capacity of the formulations. Further studies employing complementary antioxidant assays would provide a more detailed antioxidant profile of the investigated formulations.

Sample-specific blanks were used to correct for the baseline intrinsic absorbance of the beetroot extracts and gel formulations at 517 nm. This correction reduces the contribution of inherent sample colour to the measured DPPH signal. However, because colored phytochemicals may themselves participate in the DPPH reaction and undergo spectral changes during the assay, residual matrix-related spectral effects cannot be entirely excluded. Accordingly, the relatively low DPPH inhibition values should be interpreted with appropriate caution.

Although cleansing gels are rinse-off products with a relatively short contact time with the skin, this does not necessarily preclude a skin-relevant effect of antioxidant constituents. Previous studies have demonstrated that active compounds can be deposited on the stratum corneum during cleansing and remain detectable after rinsing. In particular, a single application of an α-tocopherol-containing rinse-off formulation was shown to increase vitamin E levels in skin surface lipids and to reduce UVA-induced squalene photooxidation [75]. These findings indicate that antioxidant constituents delivered from rinse-off formulations may retain biological relevance when they remain associated with the skin surface after washing. In the present study, however, deposition or retention of beetroot-derived antioxidants on the skin was not investigated. Therefore, the observed DPPH response should be interpreted as a formulation-level radical-scavenging response rather than as direct evidence of antioxidant protection of the skin.

## 3. Materials and Methods

### 3.1. Plant Material

Three cultivars of *Beta vulgaris* L., differing in root coloration, were used in this study: one yellow cultivar (Boldor), one white cultivar (Snow Ball), and one red cultivar (Dark Red Round). Seeds were obtained from Polish commercial suppliers: Bejo Zaden Poland Sp. z. o.o. (Ożarów Mazowiecki, Poland) for the yellow cultivar, TORSEED S.A. (Toruń, Poland) for the white cultivar, and W. Legutko (Jutrosin, Poland) for the red cultivar.

The plants were cultivated from June to September 2021 under open-field conditions, without the use of chemical pesticides. Beetroot roots were harvested manually at full maturity, cleaned of soil residues, rinsed with distilled water, and prepared for subsequent laboratory analyses.

### 3.2. Reagents and Reference Compounds

For the semi-quantitative analysis of saponins, reference standards of soyasaponin Bb (purity > 94%) and digoxin (purity > 98%) were purchased from Sigma-Aldrich (St. Louis, MO, USA). Soyasaponin Bb was used as the primary reference standard, while digoxin was used as an alternative reference standard for selected analyses. HPLC-grade acetone was obtained from Avantor Performance Materials Poland S.A. (Gliwice, Poland), while LC–MS-grade acetonitrile and formic acid (purity ≥ 98%) were supplied by Sigma-Aldrich (St. Louis, MO, USA).

Raw materials used for cleansing gel preparation included Sodium Lauroyl Sarcosinate (Crodasinic™ LS30; Croda Poland Sp. z o.o., Kraków, Poland), glycerin purchased from Avantor Performance Materials Poland S.A. (Gliwice, Poland), hyaluronic acid (molecular weight range 0.05–1.0 MDa) supplied by Alfa Sagittarius (Kraków, Poland), and the preservative system phenoxyethanol and ethylhexylglycerin (Euxyl^®^ PE 9010; Schülke & Mayr GmbH, Norderstedt, Germany). Citric acid (Avantor Performance Materials Poland S.A., Gliwice, Poland) was used for pH adjustment.

### 3.3. Extract Preparation and Analysis

Extraction: Beetroot roots were cleaned to remove soil residues, rinsed with distilled water, and homogenized. Approximately 250 g of fresh plant material from each cultivar was subjected to sequential maceration in four consecutive extraction cycles at room temperature using 50% (*v*/*v*) aqueous acetone as the extraction solvent. A fresh portion of solvent was used in each extraction cycle. The combined extracts were filtered and concentrated under reduced pressure at 25 °C using a Hei-VAP Advantage rotary evaporator (Heidolph Instruments GmbH & Co. KG, Schwabach, Germany). The resulting concentrates were subsequently freeze-dried using an Alpha 1-2 freeze dryer (Martin Christ Gefriertrocknungsanlagen GmbH, Osterode am Harz, Germany). From 250 g of fresh root material, 5.5 g, 5.2 g, and 6.2 g of dry extract were obtained for Boldor, Snow Ball, and Dark Red Round, respectively, corresponding to extraction yields of 2.20%, 2.08%, and 2.48% (*w*/*w*, fresh-weight basis). Thus, extraction yields were comparable among the three cultivars.

Analysis: Chemical profiling of the saponin-rich beetroot extracts was performed using ultra-high-performance liquid chromatography coupled with charged aerosol detection and quadrupole time-of-flight high-resolution tandem mass spectrometry (UHPLC–CAD–QTOF–HRMS/MS). Chromatographic separation was carried out using an Ultimate 3000 RS UHPLC system (Thermo Fisher Scientific, Waltham, MA, USA) equipped with a Corona Veo RS charged aerosol detector (Thermo Fisher Scientific, Waltham, MA, USA) and an ACQUITY UPLC BEH C18 column (Waters Corporation, Milford, MA, USA; 1.7 µm, 100 mm × 2.1 mm).

The mobile phase consisted of water containing 0.5% (*v*/*v*) formic acid (solvent A) and acetonitrile containing 0.5% (*v*/*v*) formic acid (solvent B). A linear gradient elution was applied, increasing from 24% to 55% solvent B over 30 min. The flow rate was set to 0.4 mL/min, and the column temperature was maintained at 40 °C. The column effluent was split equally between CAD and HR-MS. The CAD detector operated with a nebulizing gas pressure of 4.3 bar and an evaporator temperature of 35 °C. Mass spectrometric detection was performed using an Impact II HD QTOF mass spectrometer (Bruker Daltonics GmbH & Co. KG, Bremen, Germany) operating in negative electrospray ionization (ESI−) mode, with a scan range of m/z 100–1500 and an acquisition rate of 5 Hz. Data-dependent MS/MS experiments were conducted using ramped collision energies. Compound annotation was based on accurate mass measurements (mass error < 5 ppm), characteristic MS/MS fragmentation patterns, and comparison with previously reported spectral data.

Semi-quantitative determination of saponins was carried out using external calibration curves constructed with soyasaponin Bb (purity > 94%) and digoxin (purity >98%) as reference standards. Because the reference compounds are not structurally identical to the individual beetroot saponins, the resulting concentrations should be regarded as semi-quantitative estimates rather than definitive absolute values. The same calibration procedure was applied consistently to all samples, allowing consistent comparative assessment among cultivars. Authentic standards of the individual betavulgarosides were not available for the present study. Soyasaponin Bb was used as the primary reference standard, whereas digoxin was used as an alternative reference standard for selected analyses.

### 3.4. Preparation of Gels

A base cleansing gel was first prepared without the addition of plant extracts. The formulation consisted of deionized water (87.5% *w*/*w*), sodium lauroyl sarcosinate (7.0% *w*/*w*), glycerin (4.0% *w*/*w*), hyaluronic acid (1.0% *w*/*w*), the preservative system phenoxyethanol and ethylhexylglycerin (FEOG, 0.5% *w*/*w*), and citric acid (quantum satis) for final pH adjustment to 5.5, when required. All ingredients were combined and mixed under continuous stirring at room temperature until a homogeneous gel was obtained.

Subsequently, the formulation was enriched with 1.0% (*w*/*w*) of saponin-rich beetroot extract. The 1.0% (*w*/*w*) extract level was selected as a practically relevant concentration for natural active extracts in cosmetic formulations, including cleansing products, as comparable use levels have been reported previously [76,77]. This concentration was applied uniformly to all three extracts to enable direct comparison under identical formulation conditions. It was selected as a screening concentration and was not established as an optimum through a concentration-dependent study. To maintain a final formulation of 100% (*w*/*w*), the proportion of deionized water was reduced accordingly from 87.5% to 86.5% (*w*/*w*). The extract was added at the final stage of preparation and gently mixed until fully dispersed. The resulting formulations exhibited a homogeneous gel consistency and cultivar-dependent coloration (Table 5).

### 3.5. Evaluation of Physicochemical and Application Properties

#### 3.5.1. pH Measurement

The pH of the prepared cleansing gel formulations was measured in triplicate using a calibrated SevenMulti S47 pH meter (Mettler-Toledo GmbH, Greifensee, Switzerland) at room temperature. 

#### 3.5.2. Foamability and Foam Stability

The foamability and foam stability of the cleansing gel formulations were evaluated using a mechanical stirring method. Briefly, each sample (initial volume: 50 cm^3^) was transferred to a graduated measuring cylinder and mechanically stirred at 1000 rpm for 1 min using an RW20 digital mechanical stirrer (IKA-Werke GmbH & Co. KG, Staufen, Germany). Immediately after stirring, the total volume of the system (liquid and foam) was measured ($V_{p}$). A second measurement was performed after 5 min ($V_{t}$) to assess foam stability.

**Foamability (*Z*)** was calculated according to the following equation:

$$Z=(\frac{V_{p}}{V_{r}})\times 100\%$$

where ***Z*** represents foamability (%), $V_{p}$ is the total volume after stirring (cm^3^), and $V_{r}$ is the initial volume of the sample (50 cm^3^).

**Foam stability** (***T***) was expressed as the percentage of the foam/system volume remaining after 5 min relative to the volume measured immediately after stirring and calculated using the following equation:

$$T=\left(\frac{V_{t}}{V_{p}}\right)\times 100\%$$

where ***T*** denotes foam stability (%), and $V_{t}$ is the total volume measured after 5 min (cm^3^), and $V_{p}$ is the total volume measured immediately after stirring (cm^3^).

#### 3.5.3. Visual Assessment of Oil Emulsification and Removal

The oil emulsification and removal performance of the formulations was evaluated using a model hydrophobic soil. Rapeseed oil (0.5 mL) containing 0.1% (w/w) Sudan III (Sigma-Aldrich, St. Louis, MO, USA) was added to 500 mL of water, followed by 5.0 g of the tested formulation. The system was manually shaken for 5 min and subsequently allowed to stand for 45 min at 40 °C. The distribution of the oily phase was then assessed visually using a predefined seven-level ordinal scale ranging from 0 to 6 (Table 6). The assay was used as a semi-quantitative comparative assessment of formulation performance.

#### 3.5.4. Rheological Analysis

The rheological behavior of the formulations was characterized using an R/S Plus rheometer (Brookfield, Middleboro, MA, USA) equipped with a C75-1 cone–plate geometry and operated using Rheo3000 software, version 2.1.1103 (Brookfield, Middleboro, MA, USA). Approximately 0.5 g of each sample was analyzed at 25 °C over a shear rate range of 1–500 s^−1^. All measurements were performed in triplicate.

#### 3.5.5. Water Dispersibility Test

Water dispersibility was evaluated by adding 0.5 g of the gel to 100 mL of deionized water obtained using a Simplicity® water purification system (Merck KGaA, Darmstadt, Germany) and stirring the mixture at 50 rpm using a C-MAG magnetic stirrer (IKA-Werke GmbH & Co. KG, Staufen, Germany) while maintaining the temperature at 37 °C. The time required to achieve uniform dispersion of the formulation was measured. Each formulation was tested in five replicates. The test was used as a comparative screening assay and was not formally validated for intra- or inter-day reproducibility.

#### 3.5.6. Surface Tension Measurement

Surface tension (ST) was measured using an STA-1 tensiometer (Sinterface Technologies, Berlin, Germany) by the du Noüy ring method. Prior to each measurement, the platinum ring was thoroughly cleaned with distilled water and flame-dried. Samples were transferred into clean glass vessels, and the ring was carefully positioned at the liquid–air interface. Surface tension values were recorded as the maximum force required to detach the ring from the liquid surface.

Measurements were performed at room temperature (25 ± 1 °C) in quadruplicate. The instrument was calibrated before sample analysis using distilled water as a reference standard (ST = 70.17 ± 0.06 mN/m).

### 3.6. Evaluation of DPPH Radical-Scavenging Activity

The DPPH radical-scavenging activity of the cleansing gel formulations and the corresponding beetroot extracts was evaluated using the 2,2-diphenyl-1-picrylhydrazyl (DPPH) assay.

For preparation of the gel samples, 1.0 g of each formulation was weighed and mixed with 5 mL of analytical-grade anhydrous ethanol (Avantor Performance Materials Poland S.A., Gliwice, Poland). The samples were placed in an ultrasonic bath for 15 min at a frequency of 50 kHz, vortexed for 1 min at speed setting 6 using a VORTEX 3 mixer (IKA-Werke GmbH & Co. KG, Staufen, Germany), and subsequently centrifuged for 10 min at 3500 rpm using a Hettich centrifuge (Andreas Hettich GmbH & Co. KG, Tuttlingen, Germany). After centrifugation, 1 mL of the supernatant was collected and used for the DPPH assay.

For comparison with the gel formulations, the corresponding beetroot extracts were tested at concentrations equivalent to the extract content in the respective gels. The extract samples were subsequently subjected to the same DPPH reaction conditions as the gel-derived supernatants.

A 0.1 mM DPPH solution was prepared using 2,2-diphenyl-1-picrylhydrazyl (DPPH; Sigma-Aldrich, St. Louis, MO, USA) in analytical-grade anhydrous ethanol (Avantor Performance Materials Poland S.A., Gliwice, Poland). In a 96-well microplate, 180 µL of the DPPH solution and 20 µL of the tested sample were added to each well. The reaction mixture was incubated for 20 min in the dark at room temperature (22 ± 1 °C). After incubation, absorbance was measured at 517 nm using an Infinite 200 PRO microplate reader (Tecan, Männedorf, Switzerland).

Because the beetroot-derived samples were intrinsically colored, a sample-specific blank was included for each gel formulation and extract. The blanks contained the corresponding sample under the same assay conditions but without DPPH and were used to correct for the baseline intrinsic absorbance of the samples at 517 nm. Blank-corrected absorbance values were used for calculation of DPPH inhibition.

A DPPH solution prepared under the same assay conditions but without the tested sample was used as the assay control. The extract-free base gel was analyzed separately as a formulation-matrix control.

DPPH radical-scavenging activity was expressed as the percentage inhibition of DPPH radicals relative to the DPPH assay control and calculated using the following equation:

*DPPH inhibition* (%) = [1 − (*A*_s_ − *A*_β_)/*A*_0_] × 100

where *A*_s_ represents the absorbance of the tested sample in the presence of DPPH, *A*_β_ represents the absorbance of the corresponding sample-specific blank without DPPH, and *A*_0_ represents the absorbance of the DPPH assay control without the tested sample.

All measurements were performed in triplicate.

### 3.7. Statistical Analysis

Data are presented as mean ± standard deviation (SD) where replicate measurements were performed; the number of replicates is specified in the corresponding methodological sections. Statistical analyses were performed using Statistica 7.1 software (TIBCO Software Inc., Palo Alto, CA, USA). For semi-quantitative saponin data, differences among cultivars were evaluated using one-way analysis of variance (ANOVA) followed by Tukey’s HSD post hoc test. Statistical significance was set at *p* < 0.05. Statistical comparisons were performed only for quantifiable results; values reported as not detected (nd) or below the limit of quantification (<LOQ) were excluded from the respective analyses.

For DPPH radical-scavenging activity, differences among beetroot cultivars were evaluated separately for the gel formulations and the corresponding extracts using one-way ANOVA followed by Tukey’s HSD post hoc test. Statistical significance was set at *p* < 0.05.

## 4. Conclusions

This study demonstrates that *Beta vulgaris* L. root extracts are a source of structurally diverse triterpenoid saponins and can modify selected physicochemical and functional properties of cleansing gel formulations. UHPLC–CAD–QTOF–HRMS/MS analysis enabled the tentative annotation of 30 triterpenoid saponins, with cultivar-dependent differences in semi-quantitatively estimated saponin abundance following the order Dark Red Round > Boldor > Snow Ball. Oleanolic acid-based saponins predominated and were among the major constituents potentially contributing to the functional properties of the extracts.

Incorporation of 1% (*w*/*w*) beetroot extracts into a surfactant gel base resulted in formulations with pH values of approximately 5.3–5.4 and modified physicochemical and functional properties. Beetroot-enriched gels showed higher oil-emulsification scores than the base gel under the applied visual assay conditions and increased foamability, indicating that incorporation of the extracts modified the functional performance of the existing surfactant system. Although surface tension remained substantially lower than that of water, incorporation of the beetroot extracts did not further decrease surface tension relative to the base formulation. Instead, the extracts modified the functional behavior of the existing surfactant system, as reflected particularly by changes in emulsification and foamability. The individual contribution of beetroot-derived saponins could not be separated from that of sodium lauroyl sarcosinate under the present experimental conditions. Rheological testing showed pseudoplastic, shear-thinning behavior, while water dispersibility measurements indicated that incorporation of the extracts altered the time required for uniform dispersion of the gels in water under the applied test conditions.

Beyond cleansing performance, the beetroot-containing formulations showed cultivar-dependent DPPH radical-scavenging responses, with the highest inhibition observed for the red beetroot formulation. These findings indicate a measurable DPPH radical-scavenging response in the formulated system but should not be interpreted as evidence of substantial antioxidant functionality or direct antioxidant protection of the skin. Natural coloration imparted by beetroot pigments may represent an additional formulation attribute; however, its long-term stability requires further evaluation.

Overall, the findings support the potential use of saponin-containing beetroot extracts as sustainable, plant-derived surfactant-supporting ingredients that can modify the foaming and emulsification performance of an existing cleansing surfactant system while showing a measurable DPPH radical-scavenging response in the formulated system. Because the formulations contained sodium lauroyl sarcosinate, the present results do not establish that the beetroot extracts can function as standalone surfactants. Nevertheless, they show potential for the development of multifunctional cleansing formulations; however, dedicated skin-compatibility testing is required to evaluate their irritation potential and dermatological compatibility.

## 5. Patent

A patent application arising from the research presented in this manuscript has been filed with the Patent Office of the Republic of Poland: “Cosmetic Composition in the Form of a Cleansing Gel”, Application No. P.456802, filed on 25 August 2026. Applicant: Cracow University of Technology. The application concerns a defined cleansing gel composition containing 0.5–1.5% (*w*/*w*) dry *Beta vulgaris* L. root extract together with specified ranges of sodium lauroyl sarcosinate, glycerin, hyaluronic acid, preservative system, citric acid, and water. The claimed concept is based on the incorporation of beetroot root extract as a multifunctional ingredient contributing to both technological and bioactive properties of the formulation. Specific beetroot cultivars and the extraction procedure are included in dependent claims. The application does not rely on a demonstrated synergistic surface-tension-lowering interaction between beetroot-derived constituents and sodium lauroyl sarcosinate. The application is currently pending.

## Figures and Tables

**Table 1:** Triterpenoid saponins tentatively annotated in *Beta vulgaris* L. root extracts by UHPLC–CAD–QTOF–HRMS/MS, including retention times (tR), molecular formulas, deprotonated molecular ions, and characteristic MS/MS fragment ions.

No.	t_R_ (min)	Saponin	Betavulgaroside	*m*/*z* (M–H)^−^_exp_	Molecular Formula	*m*/*z* from MS^2^ of (M–H)^−^
1	3.5	Act-Hex-UrA-hederagenin	VI	971.4470	C_47_H_72_O_21_	851.4415; 809.4316; 647.3803; 585.3793; 471.3475
2	3.9	Act-Hex-UrA-akebonoic acid	VIII	939.4210	C_46_H_68_O_20_	819.4163; 777.4059; 657.3637; 615.3532; 553.342; 439.3214
3	4.2	Act-Hex-Hex-UrA-oleanolic acid	V	1117.5055	C_53_H_82_O_25_	997.4947; 955.4891; 835.4529; 793.4387; 455.3524
4	4.4	Hex-Hex-Pen-UrA-oleanolic acid		1087.5312	C_53_H_84_O_23_	955.4941; 925.4764; 793.4424; 745.4167; 455.3528
5	4.5	Act-Hex-Pen-UrA-oleanolic acid	IX	1087.4962	C_52_H_80_O_24_	967.4905; 925.4800; 805.4394; 763.4250; 455.3529
6	4.5	Act-Hex-UrA-oleanolic acid		955.4545	C_47_H_72_O_20_	455.3532
7	4.7	Act-Hex-Hex-UrA-oleanolic acid		1117.5065	C_53_H_82_O_25_	455.3524
8	4.7	Act-Hex-Pen-UrA-oleanolic acid		1087.4994	C_52_H_80_O_24_	455.3529
9	4.7	Act-UrA-hederagenin		809.3950	C_41_H_62_O_16_	471.3484
10	5.0	Diox-Hex-UrA-oleanolic acid	I	953.4382	C_47_H_70_O_20_	909.4487; 793.4378; 631.3846; 569.3844; 455.3529
11	5.1	Hex-Pen-UrA-oleanolic acid		925.4801	C_47_H_74_O_18_	793.4276; 701.4271; 569.3845; 455.3536
12	5.3	Act-Hex-UrA-oleanolic acid	III	955.4543	C_47_H_72_O_20_	835.4485; 793.4380; 673.3965; 631.3851; 569.383; 455.3532
13	7.6	Pen-UrA-hederagenin		779.4229	C_41_H_64_O_14_	567.3695; 471.3486
14	7.7	UrA-hederagenin		647.3808	C_36_H_56_O_10_	471.3482
15	7.7	Act-UrA-gypsogenin		807.3819	C_41_H_60_O_16_	647.3806; 471.3483
16	7.8	Act-UrA-hederagenin	VII	809.3971	C_41_H_62_O_16_	689.3910; 647.3803; 471.3484
17	8.3	Diox-Hex-UrA-oleanolic acid		953.4385	C_47_H_70_O_20_	455.3528
18	8.5	Act-Hex-UrA-oleanolic acid	III	955.4543	C_47_H_72_O_20_	835.4485; 793.4378; 631.3855; 569.3845; 455.3527
19	8.5	Hex-Pen-UrA-oleanolic acid		925.4808	C_47_H_74_O_18_	793.4376; 701.4265; 569.3840; 455.3523
20	8.8	Act-UrA-akebonoic acid		777.3701	C_40_H_58_O_15_	539.3606; 439.3509
21	8.8	Act-Hex-UrA-oleanolic acid		955.4547	C_47_H_72_O_20_	455.3532
22	9.0	UrA-akebonoic acid		615.3538	C_35_H_52_O_9_	539.3375; 493.3320; 439.3216
23	9.2	Act-UrA-gypsogenin		807.3812	C_41_H_60_O_16_	687.3453; 645.3644; 469.3327
24	9.3	Act-Pen-UrA-oleanolic acid		925.4433	C_46_H_70_O_19_	596.3845; 455.3533
25	9.6	Act-UrA-oleanolic acid	IV	793.4007	C_41_H_62_O_15_	673.3953; 631.3849; 587.3934; 455.3512
26	9.9	UrA-oleanolic acid		631.3843	C_36_H_56_O_9_	455.3531
27	10.0	Pen-UrA-oleanolic acid		763.4267	C_41_H_64_O_13_	631.3857; 569.3839; 455.3522
28	10.3	Act-UrA-oleanolic acid	IV	793.4005	C_41_H_62_O_15_	631.3842; 455.3526
29	10.3	Diox-UrA-oleanolic acid	II	791.3851	C_41_H_60_O_15_	631.3839; 555.3687; 455.3519
30	10.6	UrA-oleanolic acid		631.3840	C_36_H_56_O_9_	455.3531

Act—acetal substituent. Diox—dioxolane substituent. Hex—hexose. Pen—pentose. UrA—uronic acid. tR—retention time.

**Table 2:** Semi-quantitative profiling of triterpenoid saponins in *Beta vulgaris* L. root extracts from three cultivars—Dark Red Round, Boldor, and Snow Ball—determined by UHPLC–CAD–QTOF–HRMS/MS.

No.	t_R_ (min)	Saponin	Betavulgaroside	*m*/*z* (M–H)^−^_exp_	*Beta vulgaris* L. Content [mg/kg DE]		
					Dark Red Round	Boldor	Snow Ball
1	3.5	Act-Hex-UrA-hederagenin	VI	971.4470	68.7 ± 5.5 ^a^	nd	31.4 ± 1.6 ^b^
2	3.9	Act-Hex-UrA-akebonoic acid	VIII	939.4210	220.0 ± 17.6	nd	nd
3	4.2	Act-Hex-Hex-UrA-oleanolic acid	V	1117.5055	59.5 ± 3.8 ^a^	nd	47.9 ± 3.8 ^b^
4	4.4	Hex-Hex-Pen-UrA-oleanolic acid		1087.5312	85.6 ± 6.8 ^a^	nd	57.5 ± 4.6 ^b^
5	4.5	Act-Hex-Pen-UrA-oleanolic acid	IX	1087.4962	121.0 ± 9.7 ^a^	nd	55.9 ± 4.5 ^b^
6	4.5	Act-Hex-UrA-oleanolic acid		955.4545	<LOQ	nd	<LOQ
7	4.7	Act-Hex-Hex-UrA-oleanolic acid		1117.5065	<LOQ	nd	nd
8	4.7	Act-Hex-Pen-UrA-oleanolic acid		1087.4994	<LOQ	nd	nd
9	4.7	Act-UrA-hederagenin		809.3950	<LOQ	nd	nd
10	5.0	Diox-Hex-UrA-oleanolic acid	I	953.4382	930.2 ± 74.4 ^a^	nd	343.8 ± 27.5 ^b^
11	5.1	Hex-Pen-UrA-oleanolic acid		925.4801	<LOQ	nd	343.8 ± 20.6
12	5.3	Act-Hex-UrA-oleanolic acid	III	955.4543	3469.4 ± 277.6 ^a^	nd	1351.6 ± 81.1 ^b^
13	7.6	Pen-UrA-hederagenin		779.4229	nd	nd	63.5 ± 3.8
14	7.7	UrA-hederagenin		647.3808	78.1 ± 6.3 ^a^	<LOQ	132.8 ± 12.0 ^b^
15	7.7	Act-UrA-gypsogenin		807.3819	<LOQ	1121 ± 89.7	nd
16	7.8	Act-UrA-hederagenin	VII	809.3971	1205.9 ± 96.5 ^a^	nd	1181.3 ± 70.9 ^a^
17	8.3	Diox-Hex-UrA-oleanolic acid		953.4385	nd	<LOQ	nd
18	8.5	Act-Hex-UrA-oleanolic acid	III	955.4543	165.0 ± 13.2 ^ab^	172 ± 13.8 ^a^	135.0 ± 12.2 ^b^
19	8.5	Hex-Pen-UrA-oleanolic acid		925.4808	78.1 ± 6.3 ^c^	648 ± 51.9 ^a^	201.8 ± 12.1 ^b^
20	8.8	Act-UrA-akebonoic acid		777.3701	78.1 ± 7.3	<LOQ	<LOQ
21	8.8	Act-Hex-UrA-oleanolic acid		955.4547	<LOQ	<LOQ	nd
22	9.0	UrA-akebonoic acid		615.3538	<LOQ	<LOQ	nd
23	9.2	Act-UrA-gypsogenin		807.3812	38.3 ± 2.7 ^a^	<LOQ	33.4 ± 2.3 ^a^
24	9.3	Act-Pen-UrA-oleanolic acid		925.4433	75.1 ± 5.3 ^b^	150 ± 12.0 ^a^	44.0 ± 3.1 ^c^
25	9.6	Act-UrA-oleanolic acid	IV	793.4007	66.9 ± 4.7 ^a^	nd	44.2 ± 3.1 ^b^
26	9.9	UrA-oleanolic acid		631.3843	nd	<LOQ	<LOQ
27	10.0	Pen-UrA-oleanolic acid		763.4267	49.5 ± 3.5 ^b^	<LOQ	1447.7 ± 101.3 ^a^
28	10.3	Act-UrA-oleanolic acid	IV	793.4005	8447.1 ± 675.8 ^a^	<LOQ	6974.0 ± 557.9 ^b^
29	10.3	Diox-UrA-oleanolic acid	II	791.3851	nd	12,989 ± 1039.1	<LOQ
30	10.6	UrA-oleanolic acid		631.3840	218.7 ± 17.5 ^a^	39 ± 3.1 ^b^	230.5 ± 18.4 ^a^
		SUM			17,455 ± 742.0 ^a^	15,119 ± 1039.5 ^b^	11,623 ± 578.9 ^c^

LOQ was determined based on the soyasaponin Bb standard (6.7 mg/kg DE) and digoxin (7.5 mg/kg DE). nd, not detected; <LOQ, below the limit of quantification. Values are presented as mean ± SD (*n* = 3). Different superscript letters (^a–c^) within each row indicate statistically significant differences among beetroot cultivars (*p* < 0.05), based on one-way ANOVA followed by Tukey’s HSD post hoc test. Statistical comparisons were performed only for quantifiable values; ND and <LOQ results were excluded from the respective analyses.

**Table 3:** Physicochemical and functional properties of the base cleansing formulation and beetroot-enriched formulations.

Parameter	Gel			
	Base	White Beetroot–Enriched	Yellow Beetroot–Enriched	Red Beetroot–Enriched
pH	6.23 ± 0.02	5.30 ± 0.02	5.39 ± 0.01	5.42 ± 0.01
Oil emulsification score (0–6)	0	6	6	6
Dispersion time in water (min)	3.40 ± 0.11	5.52 ± 0.08	5.45 ± 0.07	4.65 ± 0.64
Surface tension (mN/m)	35.34 ± 0.25	41.84 ± 0.05	39.66 ± 0.03	41.29 ± 0.07
Apparent viscosity at 52 s^−1^ (Pa·s)	0.186	0.146	0.117	0.110
Apparent viscosity at 101 s^−1^ (Pa·s)	0.111	0.088	0.071	0.068
Foamability (%)	20	60	60	60
Foam stability (%)	100	67	83	90

**Table 4:** DPPH radical-scavenging activity of beetroot gel formulations and corresponding extracts at the same concentration.

Beetroot Cultivar	Gel Formulation, DPPH Inhibition (%)	Extract, DPPH Inhibition (%)
White beetroot	3.78 ± 0.39 ^b^	2.01 ± 0.21 ^b^
Yellow beetroot	0.05 ± 0.04 ^c^	0.09 ± 0.02 ^c^
Red beetroot	8.94 ± 0.87 ^a^	6.81 ± 0.47 ^a^

Values are presented as mean ± SD (*n* = 3). Different superscript letters (^a–c^) within each column indicate statistically significant differences among beetroot cultivars (*p* < 0.05), based on one-way ANOVA followed by Tukey’s HSD post hoc test. DPPH inhibition values were calculated after correction for the baseline intrinsic absorbance of each sample using the corresponding sample-specific blank.

**Table 5:** Composition and function of the cleansing gel formulations with and without beetroot extract.

No.	Ingredient	Function	% *w*/*w* (Base Gel)	% *w*/*w* (Gel + Extract)
1	Deionized water	Solvent	87.5	86.5
2	Sodium lauroyl sarcosinate	Cleansing surfactant	7.0	7.0
3	Glycerin	Humectant	4.0	4.0
4	Hyaluronic acid	Moisturizing and thickening agent	1.0	1.0
5	Phenoxyethanol, Ethylhexylglycerin (FEOG)	Preservative	0.5	0.5
6	Citric acid	pH modifier	q.s. (to pH 5.5)	q.s. (to pH 5.5)
7	Beetroot extract (saponin-rich)	Surfactant-supporting ingredient, source of bioactive phytochemicals	–	1.0

**Table 6:** Visual scoring scale for the assessment of oil emulsification and removal.

Score	Emulsion Appearance
0	Clear oil layer or visible separation of oil droplets
1	Thick (>5 mm) continuous ring of emulsified oil
2	Continuous ring of emulsified oil, 3–5 mm thick
3	Continuous ring of emulsified oil, 1–3 mm thick
4	Thin (<1 mm) ring of emulsified oil
5	Barely visible oil ring; heterogeneous emulsion in the bulk phase
6	No oil ring; homogeneous emulsion

## Data Availability

The data presented in this study are available within the article.
